# A pilot cross-sectional investigation of chronic shame as a mediator of the relationship between subjective social status and self-rated health among middle-aged adults

**DOI:** 10.1080/21642850.2023.2268697

**Published:** 2023-10-11

**Authors:** Ellen C. McGarity-Shipley, Eun-Young Lee, Kyra E. Pyke

**Affiliations:** a Cardiovascular Stress Response Laboratory, School of Kinesiology and Health Studies, Queen’s University, Kingston, ON, Canada; b In Situ Population Health Research Group, School of Kinesiology and Health Studies, Queen’s University, Kingston, ON, Canada

**Keywords:** Shame, social class, health status, adult, cross-sectional

## Abstract

Subjective social status (SSS) is an important independent predictor of health outcomes, however, the pathways through which it affects health are poorly understood. Chronic shame has previously been suggested as a potential mechanism but this has never been investigated and the relationship between chronic shame and health is under-researched. The purpose of this pilot study was to explore whether chronic shame explains a significant portion of the association between SSS and self rated health (SRH). Two-hundred American adults aged 30–55 years were recruited via a crowd-sourcing platform and were asked to provide information on their SSS, level of chronic shame, and SRH. Chronic shame significantly mediated the relationship between SSS and SRH. This pilot study provides initial evidence that shame explains a significant portion of the relationship between subjective social status and self-rated health. These findings support the initiation of larger, longitudinal investigations into chronic shame as a mediator of the subjective social status and self-rated health relationship.

## Introduction

Subjective social status (SSS), or one's own rating of their social status relative to others, is an important measure of social status that has repeatedly been associated with mental and physical health outcomes such as self-rated health (SRH) (Adler et al., 2000; Adler et al., 2008; Kraus et al., 2013; Singh-Manoux et al., 2003; Singh-Manoux et al., 2005; Thompson et al., 2014), mortality, physical functional decline (Adler et al., 2008; Kraus et al., 2013; Singh-Manoux et al., 2005), and hypertension (Adler et al., 2008; Ghaed & Gallo, 2007). Surprisingly, these associations exist even when controlling for traditional risk factors such as health behaviors and objective social status (e.g. income, education, etc.) (Adler et al., 2008; Euteneuer, 2014; Singh-Manoux et al., 2003). Scholars have speculated that the reason SSS can explain health above and beyond objective social status (OSS) is because SSS is a more meaningful cognitive averaging of all social status indicators including OSS factors, psychological factors such as feelings of financial security and perceived job control, and social factors such as discrimination (Euteneuer, 2014). However, the pathways through which SSS is associated with health are poorly understood.

One potential pathway which has been previously suggested by Wilkinson in the status anxiety hypothesis (Wilkinson, 1999) and by Kraus and colleagues (Kraus et al., 2013), but has not yet been explored is the association between SSS and chronic shame. Chronic shame is defined as a persistent feeling of inferiority, inadequacy, and deficiency and is characterized by cognitions of chronically low self-esteem and concealment behaviors (Cook, 1988; Dolezal & Lyons, 2017). Chronic shame may be involved in the relationship between SSS and health since SSS and chronic shame have similar psychological, physiological, and health correlates including low self-esteem (Cook, 1991; Euteneuer, 2014; Kan et al., 2014; Turner, 2014), elevated levels of interleukin-6 (IL-6) which is a marker of inflammation (John-Henderson et al., 2013; Rohleder et al., 2008; Saxton et al., 2011), depression (Andrews et al., 2002; Nicklett & Burgard, 2009; Rohleder et al., 2008; Singh-Manoux et al., 2005), and SRH (Lamont, 2015; Singh-Manoux et al., 2005; Thompson et al., 2014). This suggests that chronic shame could be at least a partial mediator of the relationship between SSS and health.

While stress and negative affect have previously been investigated as mechanisms of the SSS and health relationship (Kraus et al., 2013; Sapolsky, 2005), these are both very general concepts (Kemeny, 2003; Watson et al., 1988). Investigating the relationship between SSS and chronic shame, a type of stress and a component of negative affect (Dickerson et al., 2004a; Kraus et al., 2013), will allow for more specific theorization on the physiological and psychological mechanisms through which SSS affects health and how the findings can be applied to improve population health. It will also improve understandings of the relationship between chronic shame and health which is extremely under-researched to date despite findings that acute shame increases levels of pro-inflammatory molecules, stress hormones, and impairs endothelial function (Dickerson et al., 2004b, 2008; Gruenewald et al., 2004, 2006; McGarity-Shipley et al., 2022; Rohleder et al., 2008), while chronic shame is associated with lower SRH among college-aged women (Lamont, 2015), and lower life expectancy among HIV/AIDS patients (Cole, 2008; Dickerson et al., 2004a). Understanding the health impacts of chronic shame is important since shaming tactics are regularly used including society-wide mistreatment and social exclusion of stigmatized groups (e.g. LGBTQ+, certain body types, skin color) and certain parenting, coaching, and teaching strategies.

The purpose of this pilot study was to initially investigate whether chronic shame is a mechanism of the SSS-health relationship among middle-aged American adults. We tested the hypothesis that chronic shame would explain a significant portion of the relationship between SSS and SRH.

## Methods

### Participants

Two-hundred American adults aged 30–55 were recruited using Prolific (www.prolific.co), an online crowdsourcing website that is used by researchers to collect data remotely (Palan & Schitter, 2018; Peer et al., 2017). All data was collected at one time point. The sample size was based on the largest sample size estimate calculated using multiple regression data from previous studies that have studied similar variables and relationships (Adler et al., 2000; Kraus et al., 2013). All procedures were approved by the Health Sciences and Afilliated Teaching Hospitals Research Ethics Board at Queen's University (approval number: 6031601).

Objective social status (OSS) was a potentially significant confounder due to its association with SSS and chronic shame (Adler et al., 2000; Adler et al., 2008; Keltner et al., 2003; Kraus et al., 2013; Singh-Manoux et al., 2003; Walker & Bantebya-Kyomuhendo, 2014). To minimize confounding by OSS, variation in OSS was limited by excluding participants who met the following criteria: a household income of $80,000 or more (Adler et al., 2000; Adler et al., 2008; Keltner et al., 2003; Kraus et al., 2013; Singh-Manoux et al., 2003; Walker & Bantebya-Kyomuhendo, 2014) (comprises about 53% of Americans and is around the median income according to 2021 U.S. census data Semega & Kollar, 2022), incomplete high school education or completion of a graduate/professional degree (Adler et al., 2000; Adler et al., 2008; Kraus et al., 2013; Singh-Manoux et al., 2003), and unemployment (Adler et al., 2008). This exclusion strategy was used rather than only using covariation since covariation is less effective when the covariate (i.e. OSS) is systematically related to the independent variable (i.e. SSS and chronic shame) (Miller & Chapman, 2001). This is also why participants who were currently smoking or had quit smoking within the past 3 months were excluded since smoking is prevalent among lower OSS groups in the U.S. (Garrett et al., 2019) and is related to SSS and shame due to its widespread stigmatization (Brown-Johnson et al., 2015; Evans-Polce et al., 2015; Reitzel et al., 2014). Since the data was collected online using a crowdsourcing website for collecting data (Prolific), only participants who had 95% or more of their past survey submissions approved by other researchers using Prolific were allowed to participate in the study to facilitate acquisition of high quality data (Robinson et al., 2019).

Participants who were eligible for the study according to the criteria above were able to access the online Qualtrics Survey platform (Seattle, Washington, U.S.) where the study survey was hosted (participants who were ineligible did not have access to the study link). Participants were then presented with a letter of information and consent form which did not explicitly state the hypothesized relationships between variables to reduce the risk of social desirability bias. Once participants consented to the study, they were presented with several scales in the order listed below. Out of the 208 participants who consented to the study, 200 participants completed the study. Only the 200 participants who completed the study were included in analysis.

### Measures

#### Attention checks

At survey start, participants were asked to complete an attention check to improve attention to the questions (Hauser & Schwarz, 2015; Oppenheimer et al., 2009). According to the guidance of previous researchers, (Hauser & Schwarz, 2015; Oppenheimer et al., 2009) it asked participants to answer a multiple-choice question with a specific answer: ‘It is important that you carefully read the instructions before answering each question. If you understand, please choose “3” below’. A scale of 1 to 9 was presented below. Another attention check took place half-way through the study questions. Participants who failed the second attention check had their data excluded from the study (Hauser & Schwarz, 2015; Oppenheimer et al., 2009). Out of the 200 participants who completed the study, none failed either attention check.

#### Current negative affect

Before SRH was assessed, participants were asked to rate their current level of negative affect since Kraus and colleagues found that negative emotions influence SRH assessments (Kraus et al., 2013). Current negative affect was assessed using the previously validated negative subscale of the Positive and Negative Affect Schedule (PANAS) which measures 10 different negative emotions (e.g. ‘hostile’) on a 5-point Likert scale (i.e. ‘1: Very slightly or not at all’ – ‘5: Extremely’) (Watson et al., 1988). The item ‘ashamed’ was removed from this scale since shame is of particular interest in this study and was measured using a different scale (*α *= .92). All values were added and divided by 9 to result in a score ranging from 1 to 5.

#### Chronic negative affect

Chronic negative affect was assessed using the same previously validated PANAS scale as for current negative affect (Kraus et al., 2013; Watson et al., 1988). Participants were asked to indicate how strongly they had felt each emotion in the past few months (*α *= .93) to match the time of the chronic shame scale. All values were added and divided by 9 to result in a score ranging from 1 to 5.

#### Chronic stress

The previously validated Perceived Stress Scale (PSS) was used to assess chronic stress (Cohen et al., 1983). The scale included 10 items such as, ‘in the last month, how often have you been upset because of something that happened unexpectedly’. To capture a more chronic experience of stress and to match the time period of the chronic shame scale, the scale was adjusted to begin the items with ‘in the past few months’ instead of ‘in the last month’ (*α *= .90). Participants ranked each item on a 5-point Likert scale ranging from 0 (‘Never’) to 4 (‘Very Often’). All values were reverse coded where appropriate, added, and divided by 10 to result in a score ranging from 0 to 4.

#### Chronic shame

The 9-item shame subscale of the Trait Shame and Guilt Scale (TSGS) was used to assess chronic shame. The TSGS is a modification of the previously validated State Shame and Guilt Scale (SSGS) (Marschall et al., 1994) which was used in a study by Rohleder and colleagues and is the only measure of chronic shame to our knowledge (Rohleder et al., 2008). The TSGS asks participants to rate how they have felt in the past few months rather than just current feelings. For example, participants are presented with statements such as ‘I’ve wanted to sink into the floor and disappear’ which is ranked on a 5-point Likert scale ranging from 1 (‘Have not felt this way at all’) to 5 (‘Have felt this way very strongly’) (*α *= .92). All values were added and divided by 9 to result in a score ranging from 1 to 5.

#### Self-rated health (SRH)

SRH was measured using the statement, ‘In general, my health is … ’, and participants were provided with a 5-point Likert scale ranging from 1 (‘poor’) to 5 (‘excellent’). This is a widely used measure and is an independent predictor of mortality (Burström & Fredlund, 2001; Idler & Angel, 1990; Idler & Benyamini, 1997; Mossey & Shapiro, 1982).

#### Number of health conditions

Participants were also asked if a medical professional had diagnosed them with any of the following conditions: type 2 diabetes, heart attack, cancer, coronary heart disease, stroke, and chronic pain. These specific medical conditions were chosen since self-reported medical history via questionnaire has been found to be most accurate for serious conditions requiring hospitalization such as diabetes, heart attack, and cancer (Bergmann et al., 2004). Participants were also given an option to list additional conditions. This variable was represented by number of conditions (either ‘0’ or ‘≥1 condition’) to avoid violating normality assumptions.

#### Health behaviors

Participants were asked about several health-related behaviors including drinking alcohol, cigarette smoking history, use of illicit drugs, drug addiction, and leisure time physical activity (LTPA) since these variables have been previously accounted for in other studies examining the relationship between SSS and SRH (Adler et al., 2000; Singh-Manoux et al., 2003; Singh-Manoux et al., 2005). Thinking back to the past year, they were asked to report the average number of drinks they consumed in a week (collected as continuous variable), the number of times per week that they consume illicit drugs (collected as continuous variable), if they struggled with drug addiction (collected as ordinal variable: no, unsure, or yes), and the average number of minutes of leisure-time physical activity (collected as continuous variable). They were also asked if they used to smoke cigarettes and if so, they were asked when they quit, how many cigarettes they smoked per day, and for how many years to calculate pack years (calculated as continuous variable). Each of these variables was represented as a binary variable in the final analysis (see Supplementary Table 1) to avoid violating normality assumptions, except for LTPA since representing this as a binary variable significantly changed one of the results of the study in sensitivity analysis.

#### Objective social status (OSS)

OSS was assessed by household annual income, education, and employment and coded as follows. Household annual income before tax: 1 = less than $25,000; 2 = $25,000–$49,999; 3 = $50,000–$79,999 (Kraus et al., 2013). Highest education attained: 1 = graduated high school; 2 = completed community college degree; 3 = completed undergraduate degree (Kraus et al., 2013). For employment, participants were asked to report their job title and briefly describe their field and responsibilities. This variable was coded as follows using the Duncan Socioeconomic Index: 1 = blue collar (service jobs); 2 = clerical (administrative); 3 = white collar (professional) (Duncan, 1984 Ghaed & Gallo, 2007;).

#### Subjective social status (SSS)

Participants rated their national SSS using the standard MacArthur Scale of Subjective Social Status on a scale from 1 to 10 using a picture of a 10-rung ladder representing the range of social statuses in America (Adler et al., 2000; Singh-Manoux et al., 2005), with those at the top (bottom) of the ladder being the best (worst) off and having the most (least) education, money, and best (worst) respected jobs. Participants were also asked to rate their SSS within their community based on their own definition of community (Euteneuer et al., 2012; Goodman et al., 2001) since previous studies have found differences in the relationship between national SSS and health vs. community SSS and health (Cooper et al., 2010; Euteneuer, 2014).

### Statistical analysis

All analyses were performed with Stastical Package for Social Sciences (SPSS) version 27. Descriptive statistics were run to assess sample characteristics, check analysis assumptions, and check for missing data. Pariticipants with missing data were excluded from all analyses. Internal consistency of all multi-item scales was calculated and can be found in Supplementary Table 1. Before mediation analysis was conducted, bivariate Pearson correlation analysis was used to determine if there were significant correlations between: 1) national/community SSS and SRH; 2) national/community SSS and chronic shame, and; 3) chronic shame and SRH. The SSS and SRH ordinal variables were used in correlation and regression analyses since they were all normally distributed (Norman, 2010; Robitzsch, 2020). Statistical significance was defined as *p < .*05. Strength of correlations were determined as follows: small = .1–.3; medium = .3–.5; large = .5–1.0 (Cohen, 1988).

Parallel mediation analyses (Baron & Kenny, 1986) were then run using the PROCESS macro and the regression equations below where e is error (Hayes, 2022). Separate analyses were performed with national SSS or community SSS as the SSS variable. For sensitivity analysis, all statistical analyses were completed with and without non-normally distributed covariate variables being grouped into binary variables [ethnicity (0 = White; 1 = not White), alcohol use (0 = zero drinks per week; 1 = one or more drinks per week), illicit drug use (0 = zero times per week; 1 = one or more times per week), drug addiction (0 = no addiction; 1 = addiction or unsure), cigarette use (0 = never; 1 = past), LTPA (0 = <150 minutes/week; 1 = 150 minutes/week or more), and number of health conditions (0 = no health conditions; 1 = 1 or more health conditions)]. All of the previously stated binary variables were used except for LTPA which was represented as a continuous variable since representing it as a binary variable significantly changed one of the study results.

#### SSS predicting chronic shame

$$\begin{array}{rl} Chronic\;shame={\text{β}}_{0}+{\text{β}}_{1}age+{\text{β}}_{2}gender+{\text{β}}_{3}ethnicity+{\text{β}}_{4}employment\\ & +{\text{β}}_{5}education+{\text{β}}_{6}income+\\ & {\text{β}}_{7}drug\;addiction+{\text{β}}_{8}cigarette\;use+{\text{β}}_{9}alcohol\;use+{\text{β}}_{10}illicit\;drug\;use+{\text{β}}_{11}LTPA+{\text{β}}_{12}number\;of\;\\ & health\;conditions+{\text{β}}_{13}current\;negative\;affect+{\text{β}}_{14}SSS+e \end{array}$$

#### SSS predicting SRH without adjustment for chronic shame

$$\begin{array}{rl} SRH & ={\text{β}}_{0}+{\text{β}}_{1}age+{\text{β}}_{2}gender+{\text{β}}_{3}ethnicity+{\text{β}}_{4}employment+{\text{β}}_{5}education+{\text{β}}_{6}income+{\text{β}}_{7}drug\\ & addiction+{\text{β}}_{8}cigarette\;use+{\text{β}}_{9}alcohol\;use+{\text{β}}_{10}illicit\;drug\;use+{\text{β}}_{11}LTPA+{\text{β}}_{12}number\;of\;health\;\\ & conditions+{\text{β}}_{13}current\;negative\;affect+{\text{β}}_{14}SSS+e \end{array}$$

#### SSS predicting SRH with adjustment for chronic shame

$$\begin{array}{rl} SRH & ={\text{β}}_{0}+{\text{β}}_{1}age+{\text{β}}_{2}gender+{\text{β}}_{3}ethnicity+{\text{β}}_{4}employment+{\text{β}}_{5}education+{\text{β}}_{6}income+{\text{β}}_{7}drug\\ & addiction+{\text{β}}_{8}cigarette\;use+{\text{β}}_{9}alcohol\;use+{\text{β}}_{10}illicit\;drug\;use+{\text{β}}_{11}LTPA+{\text{β}}_{12}number\;of\;health\;\\ & conditions+{\text{β}}_{13}current\;negative\;affect+{\text{β}}_{14}chronic\;shame+{\text{β}}_{15}SSS+e \end{array}$$

Since chronic negative affect is associated with SRH (Kraus et al., 2013), the same regression analyses were performed again while adding chronic negative affect as a covariate to determine if the association between shame and SRH is independent of chronic negative affect. Since shame is thought of as a specific form of stress (Dickerson et al., 2004a; Kemeny et al., 2004), chronic stress was planned to be added as a covariate to see if chronic shame provides unique information. However, chronic stress and shame were found to be strongly correlated (*r =* .82, *p <* .001) and chronic stress was not added to the models to avoid violating the multicollinearity assumption.

## Results

### Participant characteristics

Participant demographic information is displayed in Table 1 (also see Supplementary table 1 for health, psychosocial scale, and health behavior information). No participants failed the attention checks and there was no missing data. For the sensitivity analysis, all results remained the same when analyses were completed with binary variables for non-normally distributed covariates (ethnicity, alcohol use, illicit drug use, drug addiction, cigarette use, LTPA, and number of health conditions) with the exception of LTPA. Results of regression analyses presented in Table 3 and supplementary tables 2 and 3 use the original continuous LTPA variable and binary variables for all other covariates.

**Table 1.** Participant characteristics.

Variable	*M* (*sd*)	% (*n*)
Age (years)	37.5 (6.9)	–
Gender		
Men (=1)	–	53.5 (107)
Women (=2)	–	45 (90)
Non-binary (=3)	–	1.5 (3)
Ethnicity		
White	–	82.5 (165)
Latinx	–	6.5 (13)
Asian	–	4.5 (9)
Black	–	4 (8)
Another	–	2.5 (5)
Ethnicity (binary)		
White (=0)	–	82.5 (165)
Not white (=1)	–	17.5 (35)
Annual income		
Less than $25,000 (=1)	–	11.5 (23)
$25,000–49,999 (=2)	–	40.5 (81)
$50,000–79,999 (=3)	–	48 (96)
Highest education completed		
High school (=1)	–	26.5 (53)
Community college degree (=2)	–	21 (42)
College degree (=3)	–	52.5 (105)
Employment		
Blue collar (service) (=1)	–	15 (30)
Clerical (administrative) (=2)	–	36 (72)
White collar (professional) (=3)		49 (98)

*n* = 200.

### Correlation analysis

Before mediation analyses were conducted, bivariate correlation analysis was used to determine whether national and community SSS were associated with chronic shame and SRH, and whether chronic shame was associated with SRH (Table 2). Both SSS variables were significantly positively associated with increased SRH (National: *r =* .29, *p <* .001; Community: *r =* .27, *p <* .001). Increased chronic shame was significantly correlated with decreased SRH (*r* = −.33, *p <* .001) and decreased SSS (National: *r =* −.37, *p <* .001; Community: *r =* −.43, *p <* .001).

**Table 2.** Correlation and unadjusted regression analyses between subjective social status (SSS), chronic shame, and self-rated health (SRH).

Predictor variable	Outcome variable	Correlation (*r*)	Regression
National SSS	SRH	.29***	*F =* 18.7***
			β = **.29** (.16, .43)***
Community SSS	SRH	.27***	*F =* 15.6***
			β = **.27** (.14, .41)***
National SSS	Chronic shame	−.37***	*F =* 31.9***
			β = −**.37** (−.50, −.24)***
Community SSS	Chronic shame	−.43***	*F =* 44.5***
			β = −**.43** (−.56, −.30)***
Chronic shame	SRH	−.33***	*F =* 24.9***
			β = −**.33** (−.47, −.20)***

Numbers in brackets represent standardized beta confidence intervals.

* *p <* .05. ** *p <* .01. *** *p <* .001. *n =* 200.

### Investigating chronic shame as a mediator between SSS and self-rated health

After initial bivariate correlation analysis confirmed that national and community SSS were associated with chronic shame and SRH, and that chronic shame was associated with SRH, mediation analysis was conducted with all covariates (Table 3 and Supplementary tables 2 and 3). It was hypothesized that chronic shame would significantly mediate the relationship between each SSS variable and SRH. In the analysis using national SSS, the association between SSS and chronic shame was statistically significant (*t(185) =* −3.75, *p <* .001) as well as the association between chronic shame and SRH (*t(184) =* −2.40, *p <* .05). When chronic shame was accounted for, the previously statistically significant relationship between SSS and SRH (*t*(185) =2.22, *p* < .05) was reduced in strength and became statistically non-significant (*t*(184) =1.53, *p* = .13).

**Figure 1.**
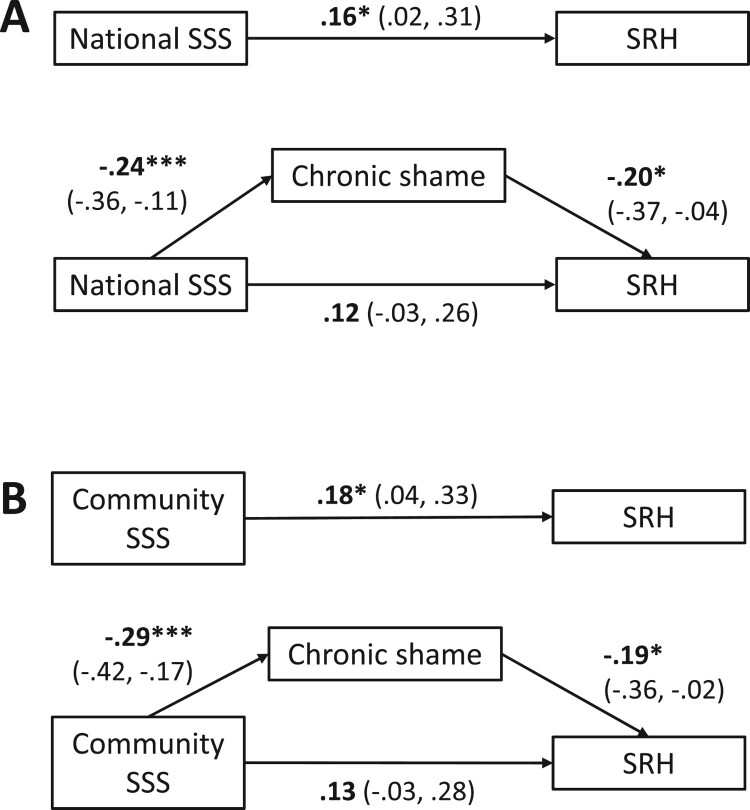
Depiction of the relationship between subjective social status (SSS) and self-rated health (SRH) mediated by chronic shame (Panel A: national SSS; Panel B: community SSS). Model controls for age, gender, ethnicity, employment, education, income, drug addiction, cigarette use, alcohol use, illicit drug use, leisure time physical activity, number of health conditions, and current negative affect. Chronic shame explained a significant portion of the relationship between each of the SSS variables and SRH. These results did not change when chronic negative affect was added to the model as a covariate. Numbers represent standardized beta coefficients (95% confidence intervals obtained through bootstrapping). *p<.05. ***p<.001. *n=*200.

The PROCESS bootstrapping procedure with 5000 resamples found that the 95% confidence interval of the standardized indirect effect of SSS on SRH through chronic shame was between .0049 and .1074. Since this confidence interval did not include 0, this suggests that SSS significantly affects SRH through chronic shame (Figure 1). Based on the proportion that the indirect effect represented (.0481) relative to the total effect of SSS on SRH (.1631), 29% of the effect that SSS has on SRH can be accounted for through chronic shame. When community SSS was used as the predictor, the results did not significantly change except the relationship between community SSS and SRH before chronic shame was added to the model was non-significant when the binary LTPA variable was used (continuous LTPA variable used in all regression analyses instead). These results did not significantly change with the addition of chronic negative affect as a covariate.

## Discussion

This pilot study was the first to explore whether chronic shame explains a significant portion of the association between subjective social status (SSS) and self-rated health (SRH). The present study found that chronic shame significantly mediated the relationship between SSS and self-rated health.

Scholars have previously hypothesized that shame may be on the causal pathway between SSS and health (Kraus et al., 2013; Wilkinson, 1999) including Wilkinson who theorized that the reason low SSS impacts health is because it is associated with feelings of inferiority and emotional distress, or in other words, shame. The present study was the first to investigate this hypothesis and found that chronic shame explained a significant portion of SSS's relationship with SRH after controlling for demographic, socioeconomic, health behavior, and health condition variables. This supports Wilkinson's status anxiety hypothesis that shame helps to explain the relationship between SSS and health (Wilkinson, 1999) and extends the findings of Kraus and colleagues (Kraus et al., 2013) on chronic negative affect partially explaining the relationship between SSS and SRH. Chronic negative affect is a general construct which is a composite of many different emotions or affectual states, including shame (Watson et al., 1988). To fully understand how SSS impacts health, it is important to investigate how specific emotions in the negative affect scale are involved. Since the present study found that shame is a mediator of the SSS – SRH relationship despite controlling for chronic negative affect (shame item removed) and chronic negative affect was no longer related to SRH after controlling for chronic shame, chronic shame may be one of the main emotions explaining why chronic negative affect has previously been found as a mediator of the SSS – SRH relationship (Kraus et al., 2013).

It is likely that the health associations of chronic shame and negative affect are difficult to tease apart since they are highly associated with each other despite exclusion of the shame item from the negative affect scale (*r =* .66, *p <* .001). Further research is needed to determine how these different emotions interact together to affect health and what other items on the negative affect scale may be particularly important determinants of health. While not a specific hypothesis of this study, a strong correlation was found between chronic shame and stress (*r =* .82, *p <* .001). This strong correlation between the two constructs adds support to the theory that shame and stress are highly related, with shame likely being a specific form of stress (Dickerson et al., 2004a; Kemeny et al., 2004). However, continuing to specifically investigate chronic shame rather than only chronic stress is a valuable endeavor since stress is a general concept (Kemeny, 2003) and may have less utility in theorizing on the specific psychological and physiological mechanisms linking SSS and health, and in the development of meaningful strategies to influence population health such as the destigmatization of marginalized groups. Further investigation is required to determine whether a distinct influence of shame vs. stress in general can be determined for a range of health outcomes.

Previous work by Lamont and colleagues (Lamont, 2015) found that chronic body shame predicted SRH in college-aged women. The present study findings support and extend this work by demonstrating that a more general measure of chronic shame also predicts SRH among middle-aged American adults. This relationship between chronic shame and SRH existed even after demographic, socioeconomic, health behavior, health condition variables, and SSS were accounted for (Table 3). In conjunction with the findings of Lamont and colleagues (Lamont, 2015), the findings of this study suggest that chronic shame is an independent predictor of SRH which is an important health measure that independently predicts mortality (Burström & Fredlund, 2001; Idler & Angel, 1990; Idler & Benyamini, 1997; Mossey & Shapiro, 1982). While research on the physiological correlates of shame that could explain its relationship with SRH is limited, shame-associated elevations in pro-inflammatory molecules and stress hormones could be involved (Dickerson et al., 2004b, 2008; Gruenewald et al., 2004, 2006; Rohleder et al., 2008). Overall, these results indicate that shame could be an important and overlooked determinant of health, and further studies are needed to better understand the full breadth of impact that shame has on health and the physiological mechanisms explaining these impacts.

**Table 3.** Regression analyses investigating chronic shame as a mediator of the relationship between national subjective social status (SSS) and self-rated health (SRH).

Predictor variables	SRH (β)
**Unadjusted for chronic shame**	
Overall model	***F****=* 3.61***
**National SSS**	**.16** (.02, .31)*
Current negative affect	−**.20** (−.33, −.06)**
Number of health conditions	−**.12** (−.26, .02)
LTPA	**.16** (.03, .29)*
Illicit drug use	**.06** (−.10, .23)
Alcohol use	−**.06** (−.19, .07)
Cigarette use	−**.02** (−.16, .12)
Drug addiction	−**.01** (−.16, .15)
Income	**.19** (.04, .34)*
Education	**>.01** (−.14, .14)
Employment	**>**−**.01** (−.14, .13)
Ethnicity	−**.02** (−.15, .11)
Gender	−**.07** (−.20, .06)
Age	−**.05** (−.19, .09)
**Adjusted for chronic shame**	
Overall model	***F****=* 3.84***
**National SSS**	**.12** (−.03, .26)
**Chronic shame**	−**.20** (−.37, −.04)*
Current negative affect	−**.10** (−.26, .05)
Number of health conditions	−**.12** (−.26, .02)
LTPA	**.16** (.03, .29)*
Illicit drug use	**.07** (−.09, .23)
Alcohol use	−**.06** (−.19, .07)
Cigarette use	−**.02** (−.16, .12)
Drug addiction	−**.01** (−.16, .15)
Income	**.16** (.01, .31)*
Education	**.01** (−.13, .15)
Employment	−**.02** (−.15, .12)
Ethnicity	−**.01** (−.14, .12)
Gender	−**.07** (−.20, .06)
Age	−**.07** (−.21, .07)

Numbers represent standardized beta coefficients (β) and standardized beta confidence intervals. Models with community SSS instead of national SSS can be found in supplementary table 3.

LTPA=leisure time physical activity.

* *p <* .05. ** *p <* .01. *** *p <* .001. *n =* 200.

### Limitations

One of the main limitations of the present study is the small sample size. Since there are no datasets to our knowledge that include measurement of SSS, SRH, and a previously validated measure of chronic shame, we collected our own data for this study and sample size calculations revealed a sample size of 200 would be adequate to answer the study question. However, due to the small sample size, we do believe that the results of the present study should be interpreted as initial pilot findings, and studies with larger sample sizes will need to be conducted to further investigate the role of chronic shame in the association between SSS and SRH.

The diversity of this sample in terms of socioeconomic status, age, country of residence, and health behaviors was largely restricted to limit confounding factors that may covary with shame and health. Despite the study being open to all racial communities, 82.5% of the study sample identified as white. The lack of racial diversity in the study sample was likely partially due to the use of a crowdsourcing website (Prolific) for recruitment which has previously demonstrated low racial diversity (Peer et al., 2017). In addition, the lack of diversity of socioeconomic status in the present sample means that the relationships found in this initial pilot study cannot be assumed to be present for a sample with a wider range of socioeconomic status levels. This limits the generalizability of the study results to other racial and socioeconomic status groups and further research should be conducted to determine if the relationships found in the present study exist among different racial groups, as well as groups that differ in socioeconomic status, age, country of residence, and health behaviors.

Several measures were taken to circumvent the potential pitfalls of recruiting via a crowdsourcing website. Only participants with a high Prolific submission approval rating were able to complete the study (Robinson et al., 2019), two different attention checks were included in the survey to assess whether participants were fully reading the item instructions before answering (Oppenheimer et al., 2009), and current levels of negative affect were controlled for in statistical analyses since emotional state can influence SRH (Kraus et al., 2013). However, the recruitment strategy excluded people who fit the inclusion criteria of the study but did not have access to a computer or Internet. Lastly, while current negative affect was controlled for, the ways in which participants’ current emotional states may have impacted their ratings on the emotion and SRH scales could not be fully controlled for.

The findings of the present study are based on cross-sectional data, therefore, causality or direction of causality cannot be confirmed and reverse causality, such as the possibility of poor health leading to chronic shame, cannot be ruled out. Some of the scales also asked participants to rate their emotions or health over different time periods (past few months vs. in general) which may have impacted the accuracy of correlations between emotion and health variables. In addition, this study did not measure any physiological factors that might help to explain why shame is associated with health. Further longitudinal and experimental research is needed to establish causality, direction of causality, and explore the physiological correlates of shame.

## Conclusions

Subjective social status (SSS) is an important and independent predictor of health, however, the pathways through which it impacts health are not well understood. Chronic shame has been previously suggested as a mechanism of the SSS-health relationship but this was unexplored before this study and the health impacts of chronic shame are under-researched. This research provided initial evidence that chronic shame helps to explain how SSS impacts health. These findings suggest that shame may be an important determinant of health and a factor that explains the relationship between SSS and health. These findings should be interpreted as initial pilot study findings that inform and encourage further research on chronic shame as a mediator of the relationship between SSS and health.

## Supplementary Material

Supplemental Material

## Data Availability

The data that support the findings of this study are available from the corresponding author upon request.
