# Supplementary material for: A pilot cross-sectional investigation of chronic shame as a mediator of the relationship between subjective social status and self-rated health among middle-aged adults
Source: Health Psychol Behav Med. 2023 Oct 11;11(1):2268697. doi: 10.1080/21642850.2023.2268697 (PMC10569351; doi:10.1080/21642850.2023.2268697)
Supplement: Supplemental Material [file RHPB_A_2268697_SM8490.docx]

**Supplement**

Supplementary table 1

*Participant characteristics: Health, psychosocial scales, and health behaviors*

| **Variable** | **M (SD)** | **% (n)** |
| --- | --- | --- |
| SRH | 3.57 (.79) | -------- |
|  |  |  |
| Number of health conditions  *0 conditions (=0)*  *≥ 1 condition (=1)* | --------  -------- | 84.5 (169)  15.5 (31) |
|  |  |  |
| Psychosocial scales |  |  |
| National SSS | 4.70 (1.57) | -------- |
| Community SSS | 4.89 (1.62) | -------- |
| Chronic shame | 2.32 (.92) | -------- |
| Chronic negative affect | 1.85 (.77) | -------- |
| Chronic stress | 1.68 (.79) | -------- |
| Current negative affect | 1.43 (.60) | -------- |
|  |  |  |
| Health behaviors  Alcohol use  *0 drinks/week (=0)*  *≥1 drinks/week (=1)* | ----------  ---------- | 44 (88)  56 (112) |
| Cigarette use  *Never (=0)*  *Past (=1)* | ----------  ---------- | 81.5 (163)  18.5 (37) |
| LTPA (mins/week) | 224 (751) | ---------- |
| LTPA binary  *Less than 150 mins/week (=0)*  *150 or more mins/week (=1)* |  | 65 (129)  35 (71) |
| Illicit drug use  *0 times/week (=0)*  *≥ once/week (=1)* | ----------  ---------- | 96.5 (193)  3.5 (7) |
| Drug addiction  *No (=0)*  *Yes or unsure (=1)* | ----------  ---------- | 98 (196)  2 (4) |

SRH=self-rated health; SSS=subjective social status; LTPA=leisure time physical activity. *n=*200.

Supplementary table 2

*Regression analysis investigating subjective social status (SSS) as a predictor of chronic shame*

| Predictor variables | Chronic shame ($\beta$) |
| --- | --- |
| Overall model  **National SSS**  Current negative affect  Number of health conditions  LTPA  Illicit drug use  Alcohol use  Cigarette use  Drug addiction  Income  Education  Employment  Ethnicity  Gender  Age | ***F*** *=* 9.53***  **-.24** (-.36, -.11)***  **.49** (.37, .60)***  **<.01** (-.11, .13)  **<.01** (-.11, .11)  **.05** (-.09, .18)  **-.02** (-.13, .09)  **<.01** (-.11, .12)  **>-.01** (-.14, .13)  **-.12** (-.25, .01)  **.05** (-.07, .17)  **-.06** (-.18, .05)  **.02** (-.09, .14)  **<.01** (-.11, .12)  **-.11** (-.23, .01) |
|  |  |
|  |  |
| Overall model  **Community SSS**  Current negative affect  Number of health conditions  LTPA  Illicit drug use  Alcohol use  Cigarette use  Drug addiction  Income  Education  Employment  Ethnicity  Gender  Age | ***F*** *=* 10.3***  **-.29** (-.42, -.17)***  **.46** (.35, .58)***  **.01** (-.11, .13)  **-.07** (-.18, .04)  **.05** (-.09, .18)  **>-.01** (-.12, .10)  **.03** (-.09, .14)  **-.03** (-.16, .11)  **-.09** (-.22, .04)  **.04** (-.08, .16)  **-.07** (-.19, .05)  **.03** (-.08, .14)  **>-.01** (-.11, .11)  **-.12** (-.24, <-.01)* |

Numbers represent standardized beta coefficients ($\beta$) and standardized beta confidence intervals.

LTPA=leisure time physical activity.

* *p <* .05. ** *p <* .01. *** *p <* .001. *n =* 200.

Supplementary table 3

*Regression analyses investigating chronic shame as a mediator of the relationship between community subjective social status (SSS) and self-rated health (SRH)*

| Predictor variables | SRH ($\beta$) |
| --- | --- |
| **Unadjusted for chronic shame** |  |
| Overall model  **Community SSS**  Current negative affect  Number of health conditions  LTPA  Illicit drug use  Alcohol use  Cigarette use  Drug addiction  Income  Education  Employment  Ethnicity  Gender  Age | ***F*** *=* 3.71***  **.18** (.04, .33)*  **-.19** (-.32, -.05)**  **-.12** (-.26, .02)  **.21** (.07, .34)**  **.06** (-.10, .23)  **-.06** (-.20, .07)  **-.04** (-.18, .10)  **<.01** (-.15, .17)  **.17** (.02, .32)*  **.01** (-.13, .15)  **<.01** (-.13, .14)  **-.02** (-.15, .11)  **-.07** (-.20, .07)  **-.04** (-.18, .10) |
|  |  |
|  |  |
| **Adjusted for chronic shame** |  |
| Overall model  **Community SSS**  **Chronic shame**  Current negative affect  Number of health conditions  LTPA  Illicit drug use  Alcohol use  Cigarette use  Drug addiction  Income  Education  Employment  Ethnicity  Gender  Age | ***F*** *=* 3.87***  **.13** (-.03, .28)  **-.19** (-.36, -.02)*  **-.10** (-.25, .06)  **-.12** (-.26, .02)  **.19** (.06, .33)**  **.07** (-.09, .23)  **-.07** (-.20, .07)  **-.03** (-.17, .11)  **<.01** (-.15, .16)  **.15** (<.01, .31)*  **.02** (-.12, .16)  **-.01** (-.15, .13)  **-.02** (-.15, .11)  **-.07** (-.20, .06)  **-.07** (-.21, .07) |
|  |  |

Numbers represent standardized beta coefficients ($\beta$) and standardized beta confidence intervals.

LTPA=leisure time physical activity.

* *p <* .05. ** *p <* .01. *** *p <* .001. *n =* 200.
